# Lateral/basolateral amygdala serotonin type-2 receptors modulate operant self-administration of a sweetened ethanol solution via inhibition of principal neuron activity

**DOI:** 10.3389/fnint.2014.00005

**Published:** 2014-01-30

**Authors:** Brian A. McCool, Daniel T. Christian, Jonathan A. Fetzer, Ann M. Chappell

**Affiliations:** Department of Physiology and Pharmacology, Wake Forest School of Medicine, Winston-SalemNC, USA

**Keywords:** sipper model, microinjection, alpha-methyl-5-hydroxytryptamine, population spike/field excitatory postsynaptic potential, ketanserin, whole-cell patch clamp

## Abstract

The lateral/basolateral amygdala (BLA) forms an integral part of the neural circuitry controlling innate anxiety and learned fear. More recently, BLA dependent modulation of self-administration behaviors suggests a much broader role in the regulation of reward evaluation. To test this, we employed a self-administration paradigm that procedurally segregates “seeking” (exemplified as lever-press behaviors) from consumption (drinking) directed at a sweetened ethanol solution. Microinjection of the nonselective serotonin type-2 receptor agonist, alpha-methyl-5-hydroxytryptamine (α-m5HT) into the BLA reduced lever pressing behaviors in a dose-dependent fashion. This was associated with a significant reduction in the number of response-bouts expressed during non-reinforced sessions without altering the size of a bout or the rate of responding. Conversely, intra-BLA α-m5HT only modestly effected consumption-related behaviors; the highest dose reduced the total time spent consuming a sweetened ethanol solution but did not inhibit the total number of licks, number of lick bouts, or amount of solution consumed during a session. *In vitro* neurophysiological characterization of BLA synaptic responses showed that α-m5HT significantly reduced extracellular field potentials. This was blocked by the 5-HT2A/C antagonist ketanserin suggesting that 5-HT2-like receptors mediate the behavioral effect of α-m5HT. During whole-cell patch current-clamp recordings, we subsequently found that α-m5HT increased action potential threshold and hyperpolarized the resting membrane potential of BLA pyramidal neurons. Together, our findings show that the activation of BLA 5-HT2A/C receptors inhibits behaviors related to reward-seeking by suppressing BLA principal neuron activity. These data are consistent with the hypothesis that the BLA modulates reward-related behaviors and provides specific insight into BLA contributions during operant self-administration of a sweetened ethanol solution.

## INTRODUCTION

The amygdala plays a central role in the regulation of risk-reward valuation. The lateral and basolateral amygdala (BLA) subdivisions form the primary input nuclei and receive processed sensory and executive information from cortical and subcortical areas (Ottersen, 1982; Brinley-Reed et al., 1995). Large, pyramidal-shaped BLA principal neurons subsequently send glutamatergic projections throughout the extended amygdala (De Olmos et al., 1985). These projections help regulate the psychological and physiological manifestations of fear/anxiety. More recent work has shown that BLA projections to regions like the nucleus accumbens also regulate the self-administration of natural rewards (Stuber et al., 2011) as well as relapse-like drug seeking for cocaine (Lasseter et al., 2011), heroin (Rizos et al., 2005), and amphetamine (Rademacher et al., 2010). These data are consistent with the BLA establishing or evaluating risk-reward relationships and provide a much broader understanding of amygdala-dependent behavioral regulation. However, the role of the BLA with respect to reward-seeking versus consumption, particularly during operant self-administration, has not been firmly established.

Samson et al. (1999) developed a self-administration model that procedurally segregates seeking behaviors from consumption. In this model, rats are trained to complete an operant response requirement within a specific time frame to then gain time-limited access to a sipper tube connected to a liquid reward (Samson et al., 1999), most frequently a sucrose and/or ethanol-containing solution. The seeking (lever press)- and consumption-related behaviors are thus procedurally separated allowing independent assessment of their neurobiological mechanisms. Subsequent work with this model has been somewhat limited but clearly demonstrates that seeking and consumption are independently controlled by overlapping but distinct neural circuits and neurotransmitter systems (Czachowski et al., 2001; Samson and Chappell, 2004; Czachowski, 2005; Henderson and Czachowski, 2011). These findings are consistent with feeding studies showing independent but integrated neural circuits controlling seeking versus intake of food rewards (Pratt and Blackstone, 2009). BLA contributions to these distinct seeking- and consumption-related behaviors have never been examined.

In contrast to the largely positive influence dopaminergic neurotransmission has on reward seeking and consumption, serotonergic neurotransmission appears to play a complimentary inhibitory role. For example, increasing serotonin levels with reuptake inhibitors appears to decrease seeking and/or self-administration for ethanol (Haraguchi et al., 1990), cocaine (Burmeister et al., 2003), and amphetamine (Porrino et al., 1989). Conversely, serotonin depletion increases seeking for both cocaine (Loh and Roberts, 1990) and natural rewards like food (Roberts et al., 1994). Thus, serotonergic neurotransmission appears to be critical for limiting self-administration of both natural and drug rewards. In particular, Roberts and colleagues implicate serotonergic neurotransmission in the amygdala as being an important modulator of reward-seeking (Roberts et al., 1994). But, the specific neurotransmitter receptors, amygdala subdivisions, cellular populations, and neurophysiological mechanisms mediating these effects are largely unexplored.

Serotonin exerts its biological action via the activation of a heterogeneous collection of receptors that include both heterotrimeric G protein-coupled receptors and a ligand-gated ion channel. mRNAs, protein, or binding activity for the G protein-coupled 5-HT1 and 2 receptors and their various isoforms are expressed in the BLA (Bruinvels et al., 1994; Wright et al., 1995; Li et al., 2003; McDonald and Mascagni, 2007), but their neurophysiological role has not been extensively characterized. BLA 5-HT1A receptors suppress both GABAergic (Koyama et al., 2002) and glutamatergic synaptic transmission (Cheng et al., 1998). BLA 5-HT2A/C receptors have complex neurophysiological effects and appear to both stimulate BLA GABAergic interneuron activity (Rainnie, 1999; Jiang et al., 2009) and may directly inhibit BLA principal neurons (Rainnie, 1999) though the precise mechanisms governing this latter effect need to be better understood. Further, the precise behavioral roles these different BLA receptors play in the modulation of operant self-administration is not currently clear.

In the present work, we have used a self-administration paradigm that procedurally segregates seeking and consumption to examine the role of the BLA in modulating these independent behavioral components. We integrate this approach with direct BLA microinjection of a 5-HT2-selective agonist. These behavioral approaches are complimented by *in vitro* investigation of the neurophysiological impact of this agonist. Our findings provide strong support for the notion that BLA neuron activity is critical for the regulation of reward seeking-related behaviors and highlights potential cellular mechanisms that mediate these outcomes.

## MATERIALS AND METHODS

### ANIMAL SUBJECTS

Adult male Long-Evans rats (250 g) were purchased from Harlan (Indianapolis, IN, USA). Animals were subjected to handling and behavioral manipulations according to the NIH Guide for the Care and Use of Research Animals. All procedures were approved by the Wake Forest Medical School IACUC. Animals were individually housed on a standard 12 h light/dark cycle (6 am lights on) with food and water *ad libitum* except during the 2-h self-administration sessions (below).

### OPERANT SELF-ADMINISTRATION

All experiments were performed in sound-attenuated operant chambers (Med Associates, St. Albans, Vermont, USA) equipped with house lights, fans, operant levers, and sipper tubes as described previously (McCool and Chappell, 2009). Commercially available software (MedPC, Med Associates) controlled access to levers and sipper tube access. Three days prior to operant training, animals were given an initial forced exposure to 10% ethanol in the home cage during which time ethanol was the only liquid available as described (Samson et al., 2001). During this period, animals consumed 5.3 ± 0.2 g/kg ethanol per day. The subsequent operant training consisted of an initial 4 h session when rats (*n* = 9) were trained with an FR1 schedule that provided 40 s of access to sipper tubes containing a 10% sucrose solution in water. Over the next week, we gradually decreased session time and gradually increased the fixed ratio schedule and sipper tube access time until daily sessions consisted of a single 20 min period to fulfill the desired response requirement (RR) followed by a single 20 min sipper tube access-period. This operational segregation of lever-press and drinking periods allowed independent measurement of “seeking” and “consuming” behaviors (Samson et al., 2001). Self-administration sessions started 1 h after lights-on and ran 5 days each week until the end of the microinjections. Over several weeks, we increased response requirements and gradually lowered the sucrose concentration/increased ethanol concentration in the sipper until animals had to fulfill a RR30 to access as solution containing 2% sucrose/10% ethanol. We utilized sweetened ethanol to avoid potential confounds related to food- versus drug-rewards (Czachowski, 2005). Eight out of nine individuals consistently executed the RR30 for sucrose/ethanol and were included in the microinjection experiments (below). Following 2 weeks of stable responding, we subjected animals to bi-weekly, single-day “non-reinforced” sessions where levers were available but sipper tubes did not lower into the self-administration chamber. Normal RR30 reinforced sessions surrounded these “seeking” sessions the other 4 days on these weeks. Previous work has shown that, under this schedule, individual “non-reinforced” sessions produce stable lever press behaviors that do not extinguish across many weeks (Samson et al., 2001). Data from these two sessions were pooled and served as baseline “seeking” behaviors. Ethanol consumption levels during the training and baseline periods when animals were all consuming 2% sucrose/10% ethanol were 1.09 ± 0.07 g/kg per session. Ethanol intakes dropped to 0.94 ± 0.05 g/kg per session the week following the stereotaxic surgeries (see below) and remained in this range for the remainder of the study (see **Table 2**).

### SURGERIES AND MICROINJECTIONS

**FIGURE 1:**
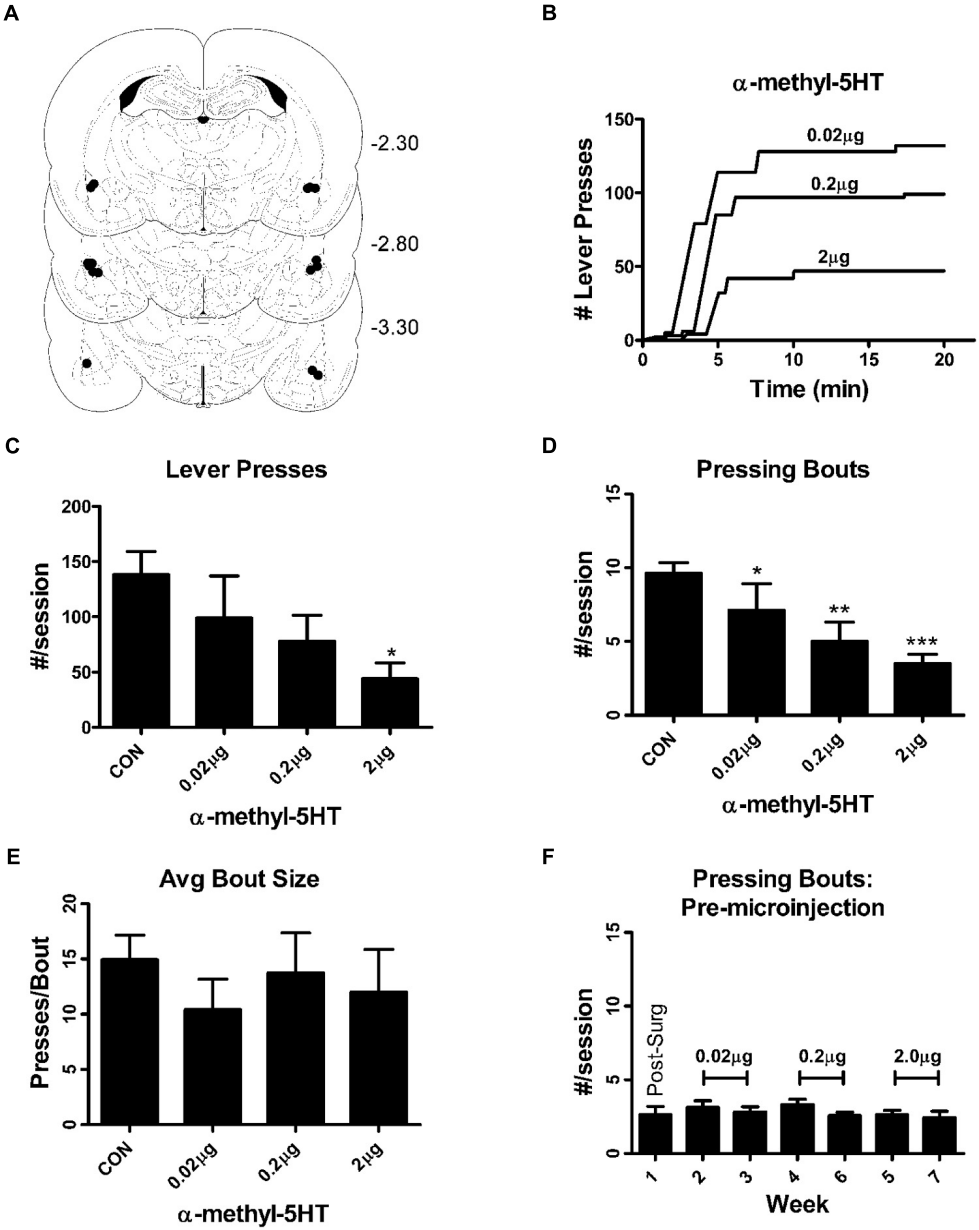
**Intra-BLA α-methy-5HT reduces seeking-like behaviors during non-reinforced self-administration sessions by reducing the total number of lever-pressing bouts.** **(A)** Representation of guide cannulae placements for the microinjection experiments. Figures are taken from Paxinos and Watson (Paxinos and Watson, 2005); numbers on the right correspond to the rostral/caudal coordinates of the coronal sections relative to Bregma. Relative placement of guide cannulae tips are indicated by the black circles (•). Injection cannulae would have extended ~0.5 mm below the guide cannulae. **(B)** Exemplars of lever-pressing behaviors from a single individual illustrating the effects microinjection of increasing doses of α-m5HT. Horizontal portions of the curves represent periods with no lever pressing. Vertical deflections represent the number of lever presses during a particular bout (here defined as continuous lever presses without a pause greater than 20 s). **(C)** Summarized data from the entire experimental group shows that intra-BLA α-m5HT significantly reduces (*P* < 0.05, repeated measures ANOVA) the total number of lever presses that occur during non-reinforced self-administration sessions. *P < 0.05 compared to pre-surgical baseline using Neuman–Keuls multiple comparisons *post hoc* test. **(D)** Intra-BLA α-m5HT reduces the total number of lever pressing bouts during non-reinforced sessions in a dose-dependent fashion. Repeated-measures ANOVA indicated significant differences (*P* < 0.001) between pre-surgical baseline and all the doses of α-m5HT used in our study. ^*^P < 0.05, ^**^P < 0.01, and ^***^P < 0.001 compared to baseline (Neuman–Keuls post-test). ^#^*P* < 0.05 compared to the 0.02 μg dose (Neuman–Keuls). **(E)** The effect of α-m5HT on lever pressing during non-reinforced sessions was not related to changes in the average number of lever presses during individual bouts (bout size). **(F)** The average number of lever pressing bouts during reinforced sessions (response requirement = 30, see Materials and Methods) on non-microinjection days was stable during the time-frame of the microinjection experiments. There were no significant effects of “week” across the experiment (repeated measures ANOVA). The doses delivered during these weeks are indicated in brackets. Additional parameters measured during the reinforced sessions (non-microinjection days) are found in **Table 1**.

Following the operant training period, animals were deeply anesthetized with pentobarbital (50 mg/kg, IP), attached to a stereotaxic frame (Kopf Instruments, Tujunga, CA, USA), and fit with chronic 26 gage guide cannula directed at the ventral aspect of the lateral amygdala [–2.80 mm from bregma; medio-lateral, from the midline +5.05 mm; ventral from the top of the brain, –6.20 mm; (McCool and Chappell, 2007)]. At the time of surgery, rats weighed 472 ± 14 g. Rats recovered over the weekend and returned to the self-administration chambers the following week. They gained weight throughout the course of the experiment (final body weight = 529 ± 18 g). Daily post-surgical handling included manipulation of the cannula obturator and exposure to microinjection pump noise. On microinjection days, Hamilton syringes delivered 0.5 μl of sterile alpha-methyl-5HT in artificial cerebrospinal fluid (see below) over 1 min through 33 gage injection cannula that extended 1.0 mm ventral from the end of the guide cannula. We left injection cannula in place for an additional minute before animals were placed into the self-administration chamber. Seeking- and consumption-related behaviors were probed on alternate weeks by microinjection of a single dose of α-m5HT prior to either a single non-reinforced session (above) or a standard self-administration session. Doses of α-m5HT were tested in an ascending series. At the end of the microinjection experiments, animals were euthanized; and brains were fixed with paraformaldehyde for post-mortem determination of cannula placement. Cannulas were placed in the target region (**Figure 1A**) in all animals included in this report. In one animal, cannulas were placed posterior of the BLA into the ventral aspect of the hippocampus (CA1). This animal was excluded from our analysis; but α-m5HT did not appear to produce any dose-dependent behavioral effects in this one subject.

### DRUGS

Alpha-methyl-5-hydroxytryptamine (α-m5HT) maleate, ketanserin tartrate, and bicuculline methiodide were purchased from Tocris Cookson (Ellisville, MO, USA). Ethanol (95%) was purchased from Warner–Graham Company (Cockeysville, MD, USA). Sucrose and salts used in the electrophysiology experiments were all purchased from Sigma–Aldrich (St. Louis, MO, USA).

### *IN VITRO* ELECTROPHYSIOLOGY

Basolateral amygdala extracellular synaptic responses and principal neuron activity were measured *ex vivo* in coronal brain slices prepared from adult male rats (Harlan) according to established procedures (Christian et al., 2013). Slices were allowed to recover for 1hr and then continuously perfused (2 ml/min) with artificial cerebrospinal fluid within a custom-built submersion-type recording chamber.

For extracellular field potential experiments, recording electrodes containing 0.9% saline measured synaptically driven “field” population spikes/excitatory postsynaptic potentials (PS-EPSP) evoked with a bipolar stimulating electrode placed along the lateral boundary of the lateral/basolateral amygdala as previously described (Lack et al., 2009). Previous work has shown this PS-EPSP is inhibited both by the voltage-gated sodium channel antagonist tetrodotoxin and by the ionotropic glutamate receptor antagonist DNQX (Lack et al., 2008; Lack et al., 2009). Stimulus intensity was adjusted in each slice so that the PS-EPSP amplitude was ~70% of maximum. In experiments were the effects of the 5-HT_2_or GABA_A_ antagonist were measured, slices were pre-incubated in antagonists prior to establishing the PS-EPSP baseline response and subsequently measuring the effect of α-m5HT. These data are therefore expressed as a percent effect and statistically compared to the non-antagonist condition.

Whole-cell current clamp recordings used an intracellular recording solution consisting of (in mM) 140 K-gluconate, 10 HEPES, 5 NaCl, 1 EGTA, 0.1 CaCl_2_, 2 Mg-ATP, 0.3 Na_2_-GTP, pH 7.2 with gluconic acid, 290 mmol/kg osmolality. Principal neurons were identified in the whole-cell configuration by their electrical properties including a large whole-cell capacitance, passive membrane properties, and action potential response to prolonged depolarizations (Rainnie et al., 1993).

Commercially available software (pClamp10.0, Molecular Devices, Sunnyvale, CA, USA) collected responses in current-clamp mode (Axoclamp 200B or Multiclamp 700B, Molecular Devices). Calibrated syringe pumps delivered α-m5HTto the recording chamber at a rate of 0.1 ml/min to produce the indicated final concentration. Concentration-effect curves were generated using an ascending concentration series. All recordings were carried out at room temperature. PS-EPSP peak (mV) and slope (mV/ms; calculated from the 20–80% rise time of the response measured from baseline to the peak of the response) and action potential threshold (mV) were measured offline.

### STATISTICAL ANALYSIS

For the microinjection experiments, we utilized a within-subject design to increase statistical power; these data were analyzed with repeated-measures one-way ANOVA and comparisons between drug doses and control values were made with Neuman–Keuls multiple-comparison post-test. Only significant differences between specific drug doses and control values are illustrated in the figures. Significant main-effects and comparisons between doses are discussed in the text. *P* values < 0.05 for both main-effect and post-test were considered significant. For the electrophysiology experiments, across-treatment comparisons were performed using standard one-way ANOVA and Neuman–Keuls multiple comparison post-tests or paired *t*-test as appropriate. *P* < 0.05 was considered significant in both cases.

## RESULTS

### INTRA-BLA α-METHYL-5HT SUPPRESSES OPERANT SUCROSE/ETHANOL SELF-ADMINISTRATION

We trained animals to lever press on a RR30 to gain 20 min access to a sweetened ethanol solution (2% sucrose/10% ethanol). Both lever responding- (**Table 1**) and consumption-related behaviors (**Table 2**) were stable on non-injection days throughout the time span of the microinjection experiments. This confirms previous findings (Samson et al., 2001) showing that the intermittent “non-reinforced” sessions used to assay seeking-like (e.g., lever press only) behaviors did not disrupt or extinguish normal operant behaviors.

**Table 1:** Responding-related behaviors across the microinjection period during non-experimental days.

	Response latency (s)^2^	Press rate (#/min)	Press time (min)
Week 1	54.3 ± 21.9	20.4 ± 6.6	4.3 ± 1.9
Week 2^1^	53.8 ± 14.1	21.0 ± 7.0	3.9 ± 1.2
Week 3	40.5 ± 6.3	24.4 ± 10.0	3.4 ± 1.3
Week 4	47.8 ± 12.5	18.7 ± 5.3	3.5 ± 1.2
Week 5	76.9 ± 19.7	24.2 ± 5.7	2.6 ± 0.9
Week 6	66.8 ± 9.6	30.4 ± 7.1	2.3 ± 0.8
Week 7	46.7 ± 16.3	25.5 ± 5.6	2.7 ± 1.1

1 0.02 μg α-methyl 5-HT was microinjected on weeks 2–3, 0.2 μg on weeks 4–5, and 2.0 μg on weeks 6–7. Values listed here represent pressing during non-injection days. Repeated measures ANOVA did not identify any significant effect of week across the parameters listed.

2 Mean weekly values were calculated using data from Monday, Tuesday, and Friday from the week just after surgery and on injection weeks (see Materials and Methods). Values are reported as mean ± SEM (*n* = 8).

**Table 2:** Consumption-related behaviors across the microinjection period during non-experimental days.

	Licks/session^2^	Amount consumed (mL)	Ethanol consumed (g/kg)	Lick bouts^3^
Week 1	839 ± 81	5.6 ± 0.5	0.91 ± 0.08	3.5 ± 0.8
Week 2^1^	889 ± 88	5.9 ± 0.5	0.92 ± 0.07	3.7 ± 1.0
Week 3	968 ± 106	6.1 ± 0.6	1.0 ± 0.08	3.8 ± 1.0
Week 4	980 ± 88	6.3 ± 0.6	0.99 ± 0.07	3.4 ± 1.1
Week 5	1123 ± 179	6.2 ± 0.5	0.95 ± 0.06	4.2 ± 1.2
Week 6	955 ± 159	5.8 ± 0.4	0.88 ± 0.06	4.4 ± 1.7
Week 7	810 ± 133	5.5 ± 0.5	0.83 ± 0.07	4.3 ± 1.4

1 0.02 μg α-methyl 5-HT was microinjected on weeks 2–3, 0.2 μg on weeks 4–5, and 2.0 μg on weeks 6–7. Values here represent drinking parameters expressed during non-microinjection days. Repeated measures ANOVA did not identify any significant effect of week across the parameters listed.

2 Mean weekly values were calculated using data from Monday, Tuesday, and Friday from the week just after surgery and on injection weeks (see Materials and Methods). Values are reported as mean ± SEM (*n* = 8).

3 A lick bout is defined as continuous touches on the sipper spout without a pause in drinking longer than 20 s.

Lever-press behaviors related to reward seeking were specifically measured during non-reinforced sessions where lever presses did not lead to presentation of the sweetened ethanol/sucrose mixture. Lever-press behaviors expressed during these non-reinforced sessions are stable for up to 7 weeks (i.e., do not extinguish) when they are separated by standard operant sessions where lever presses are reinforced with reward (Samson et al., 2001). These non-reinforced trials were specifically used to measure lever press behaviors (seeking) without potential confounds arising from the pharmacological effects of ethanol. During these non-reinforced sessions, we found that α-m5HT microinjection into the BLA significantly reduced the total number of non-reinforced lever presses (**Figures 1B,C**; *P* < 0.05, repeated measures ANOVA, *F* = 3.2, df = 7) with significant differences between the 2.0 μg dose and pre-surgery baseline (*P* < 0.05, Neuman–Keuls multiple comparison post-test). This dose-dependent reduction of non-reinforced responding was accompanied by a significant reduction in the total number of lever pressing bouts, where a bout is defined as lever pressing without any pause greater than 20 s (Samson et al., 2000), relative to the number of bouts expressed during pre-surgery baseline (**Figure 1E**; *P* < 0.001, *F* = 13.2, repeated measures ANOVA) at both the 0.2 and 2.0 μg doses (*P* < 0.01 and *P* < 0.001, respectively, Neuman–Keuls multiple comparison post-test). These effects were not due to any significant decrease in the size of individual lever pressing bouts expressed during the session (presses/bout; **Figure 1D**; *P* > 0.05, *F* = 0.5, repeated measures ANOVA) or any decrease in the total time spent responding during the session relative to control (not shown; CON = 13.5 ± 1.0 min, 0.02 μg dose = 12.0 ± 2.4 min, 0.2 mg dose = 12.3 ± 2.7 min, 2.0 μg dose = 6.6 ± 2.0 min, *P* > 0.05, *F* = 2.6, repeated measures ANOVA). But, the latency to begin responding during these non-reinforced “seeking” sessions was significantly increased (*P* < 0.001, *F* = 10.4) by BLA microinjection of the α-m5HT 2.0 mg dose (54.0 ± 9.9 s for baseline, 90.4 ± 18.0 s for 0.02 μg, 29.5 ± 10.7 s for 0.2 μg, and 123.7 ± 19.8 s for 2.0 μg; *P* < 0.01 for the later dose compared to pre-surgery). Finally, intra-BLA administration of α-m5HT did not significantly alter response rates (number of lever presses per second) during non-reinforced sessions (main effect *P* > 0.05, *F* = 1.5). To examine whether these various lever-press behaviors were stable throughout the same experimental time-frame as the microinjection experiments, we examined the standard operant sessions surrounding the non-reinforced, microinjection days. The number of bouts during these regular self-administration sessions (with RR30) did not vary across any doses (**Figure 1F**; main effect *P* > 0.05, *F* = 2.6, repeated measures ANOVA). Similarly, response latency, response rate, and total response time did not significantly vary across the entire microinjection period (**Table 1**). These findings show that intra-BLA α-m5HT significantly decreases seeking-related behaviors directed at a sweetened ethanol solution by decreasing the total number of lever pressing bouts during a session and by increasing the latency to begin lever pressing once the sessions begin.

**FIGURE 2:**
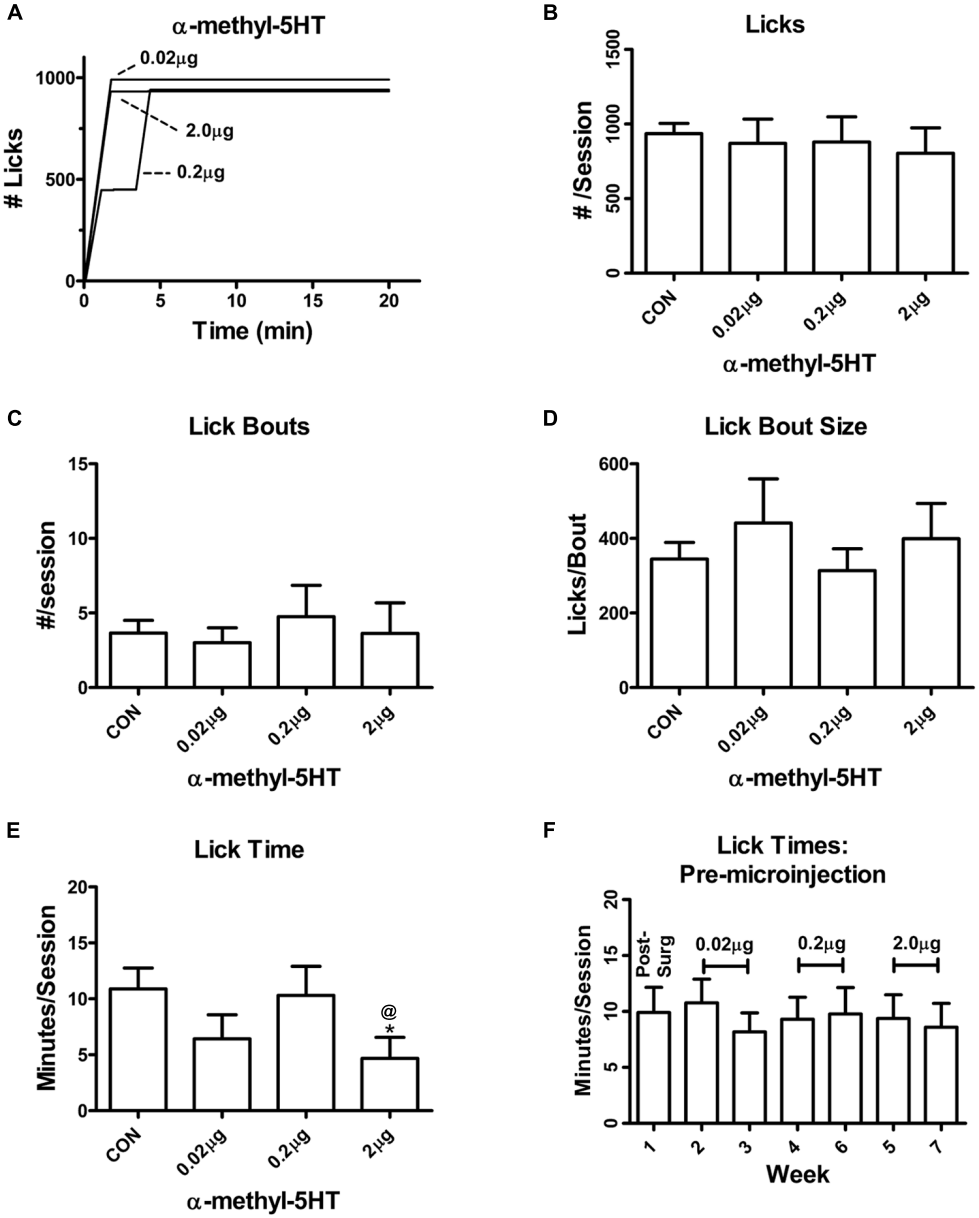
**Intra-BLA α-m5HT has modest effects on consumption related behaviors during self-administration of a sweetened ethanol solution.** **(A)** Exemplar drinking behaviors expressed by a single animal during self-administration sessions recorded following microinjection of the indicated doses. Vertical deflections indicate continuous licking (no pause greater than 20 s) of a sipper tube containing 2% sucrose/10% ethanol. Horizontal deflections show periods with no drinking. **(B)** The total number of sipper tube licks during the 20 min self administration period was not altered by microinjection of α-m5HT. There was no significant difference between pre-surgical baseline and any dose (*P* > 0.05, *F* = 0.4, repeated measures ANOVA). **(C)** Intra-BLA microinjection of α-m5HT did not alter the total number of lick bouts, defined as continuous licking of the sipper tube without a break greater than 20 s. There was no significant difference between pre-surgical baseline and any dose of α-m5HT (*P* > 0.05, *F* = 0.5, repeated measures ANOVA). **(D)** The mean number of licks per bout was not significantly altered by intra-BLA α-m5HT (*P* > 0.05, *F* = 1.0, repeated measures ANOVA). **(E)** Microinjection of 2 μg α-m5HT significantly decreased (*P* < 0.05, *F* = 4.9, repeated measures) the amount of time animals spent licking (“Lick time”) relative to both the pre-surgery baseline and the 0.2 μg dose. ^*^P < 0.05 compared to pre-surgery baseline, ^@^*P* < 0.05 compared to 0.2 μg dose, Neuman–Keuls multiple comparison post-test. **(F)** The time animals spent licking the sipper tube during the 20 min access period was stable across the entire time-frame of the microinjection experiments. There were no significant effects of “week” across the experiment (repeated measures ANOVA). The doses delivered during experimental days of these weeks are indicated in brackets. Additional drinking parameters measured during non-experimental days are found in **Table 2**.

In contrast with the intra-BLA α-m5HT suppression of lever responding during non-reinforced sessions, the 5-HT2 agonist did not appear to significantly alter most of the consumption-related behaviors in this self-administration paradigm. Neither the total number of licks directed at the sweetened ethanol solution (**Figures 2A,B**; *P* > 0.05, *F* = 0.4) nor the number of licking bouts (continuous licking without a 20 s pause; **Figures 2C,D**; *P* > 0.05, *F* = 0.4) were altered by any of the α-m5HT doses. However, we did find that the time spent licking the sipper tube was significantly reduced by BLA α-m5HTmicroinjections at the highest dose (**Figure 2E**; main effect *P* < 0.05, *F* = 4.9); and, there was also a main effect of treatment on session lick rates (CON = 159 ± 22 licks/min, 0.02 μg = 290 ± 75 licks/min, 0.2 μg = 126 ± 38 licks/min, 2.0 mg = 250 ± 62 licks/min; *P* < 0.05, *F* = 3.3) but post-tests did not identify any significant differences between specific doses. Lick times (**Figure 2F**), lick rates (not shown), and other consumption-related variables were stable on non-microinjection days across this same time period (**Table 2**). These findings appear consistent with a modest α-m5HT-dependent modulation of some drinking-related dependent variables (lick rate and lick time) but these effects were not dose- dependent.

### α-METHYL-5HT INHIBITS BLA FIELD RESPONSES *IN VITRO*

**FIGURE 3:**
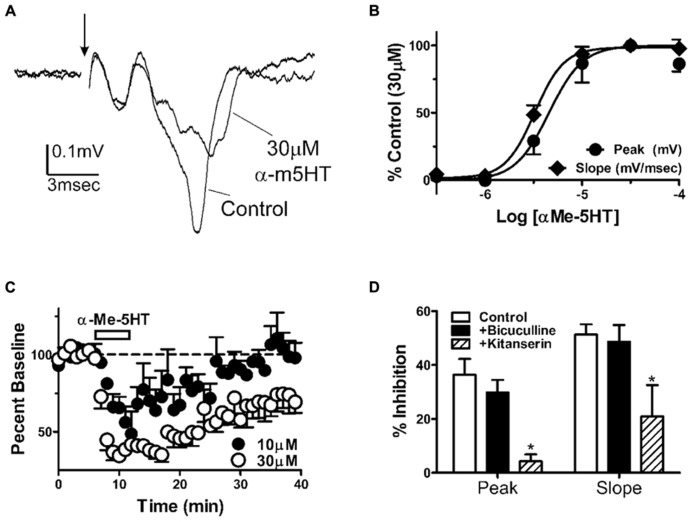
**α-m5HT inhibits BLA extracellular responses to synaptic stimulation via 5-HT2 receptors.** **(A)** Exemplar population spikes/excitatory postsynaptic potentials (PS-EPSP) evoked by stimulation of the external capsule. These extracellular responses were recorded in the BLA using an *in vitro* coronal brain slice preparation. Arrow denotes position of the stimulation artifact that has been removed for clarity. Note that 30 μM α-m5HT suppresses both the amplitude and the slope of the negative deflection that represents the BLA PS-EPSP. **(B)** Concentration-effect curves for PS-EPSP modulation by α-m5HT show that EC50 values for PS-EPSP amplitude slope (mV/ms, open circles) and amplitude (mV, closed circles) are distinct (*P* < 0.05, paired *t*-test). Data were normalized to the percent inhibition by 30 μM in each slice to reduce slice-to-slice variance in α-m5HT efficacy. **(C)** Time course of α-m5HT modulation of BLA PS-EPSP slope indicate that modulation has a rapid onset and can reverse during prolonged washout of the drug. PS-EPSP modulation appeared less robust and washed out faster with 10 μM α-m5HT (closed circles) compared to 30 μM α-m5HT (open circles). **(D)** Pharmacological analysis of α-m5HT modulation (10 μM) suggests that inhibition of the BLA PS-EPSP is mediated by 5-HT2 receptors but does not involve activation of GABAA-dependent signaling. Compared to α-m5HT modulation of the PS-EPSP alone (*n* = 10), the modulation of PS-EPSP peak amplitude and slope was significantly attenuated by 20 μM ketanserin (*P* < 0.05 and *F* = 5.8 for peak, *P* < 0.05 and *F* = 4.5 for slope, one way ANOVA, *n* = 3) but was not altered by the GABAA receptor antagonist bicuculline (10 μM, *n* = 11). *P < 0.05 compared to control from Neuman–Keuls multiple comparison post-test.

To understand the neurophysiological mechanism mediating the behavioral effects of α-m5HT microinjection into the BLA, we recorded population spikes-extracellular EPSPs (PS-EPSP) from coronal brain slices containing the BLA. We found that α-m5HT inhibited BLA PS-EPSPs in a concentration dependent manner (**Figures 3A,B**). To standardize the concentration-effect curve across slices, we normalized the effect of each concentration to the percent inhibition in the presence of 30 μM α-m5HT which appeared to be maximally efficacious in all the slices examined in this study. The effect of α-m5HT on the PS-EPSP slope (EC_50_ = 3.2 ± 1.2 μM, *n* = 7) was significantly more potent than on the peak of the response (EC_50_ = 4.6 ± 0.7 μM; *P* < 0.05, paired *t*-test). For the lower concentrations of α-m5HT (≤10 μM), inhibition was readily reversible during drug wash-out (**Figure 3C**). Although the concentration-response relationship suggests there might be some tachyphylaxis during the ascending concentration experimental design, there were no significant differences between the magnitude of PS-EPSP inhibition with the two highest concentrations (30 and 100 μM; *P* > 0.05, paired *t*-test).

To further refine the mechanisms responsible for modulation of the BLA PS-EPSP, we pretreated slices with ketanserin (20 μM), a 5-HT2A/C receptor antagonist, prior to exposure with 10 μM α-m5HT. Ketanserin significantly reduced α-m5HT inhibition of the BLA PS-EPSP response peak (**Figure 3D**; *n* = 3, *P* < 0.05 and *F* = 5.8) relative to the agonist alone (*n* = 10). These data suggest that 5HT2-like receptors mediate the neurophysiological effects of α-m5HT. The literature also suggests that 5HT2 receptors may facilitate BLA GABAergic function (Rainnie, 1999; Jiang et al., 2009); and this could be a potential mechanism governing α-m5HT-mediated suppression of the BLA PS-EPSP. However, pretreatment of BLA slices with the GABA_A_ receptor antagonist bicuculline (10 μM, *n* = 11) did not significantly alter α-m5HT modulation of the PS-EPSP (**Figure 3D**). These findings suggest that GABA_A_ receptor-mediated responses did not contribute significantly to α-m5HT inhibition of the field EPSP amplitude or slope.

### α-METHYL-5HT SUPPRESSES THE BLA PRINCIPAL NEURON EXCITABILITY

**FIGURE 4:**
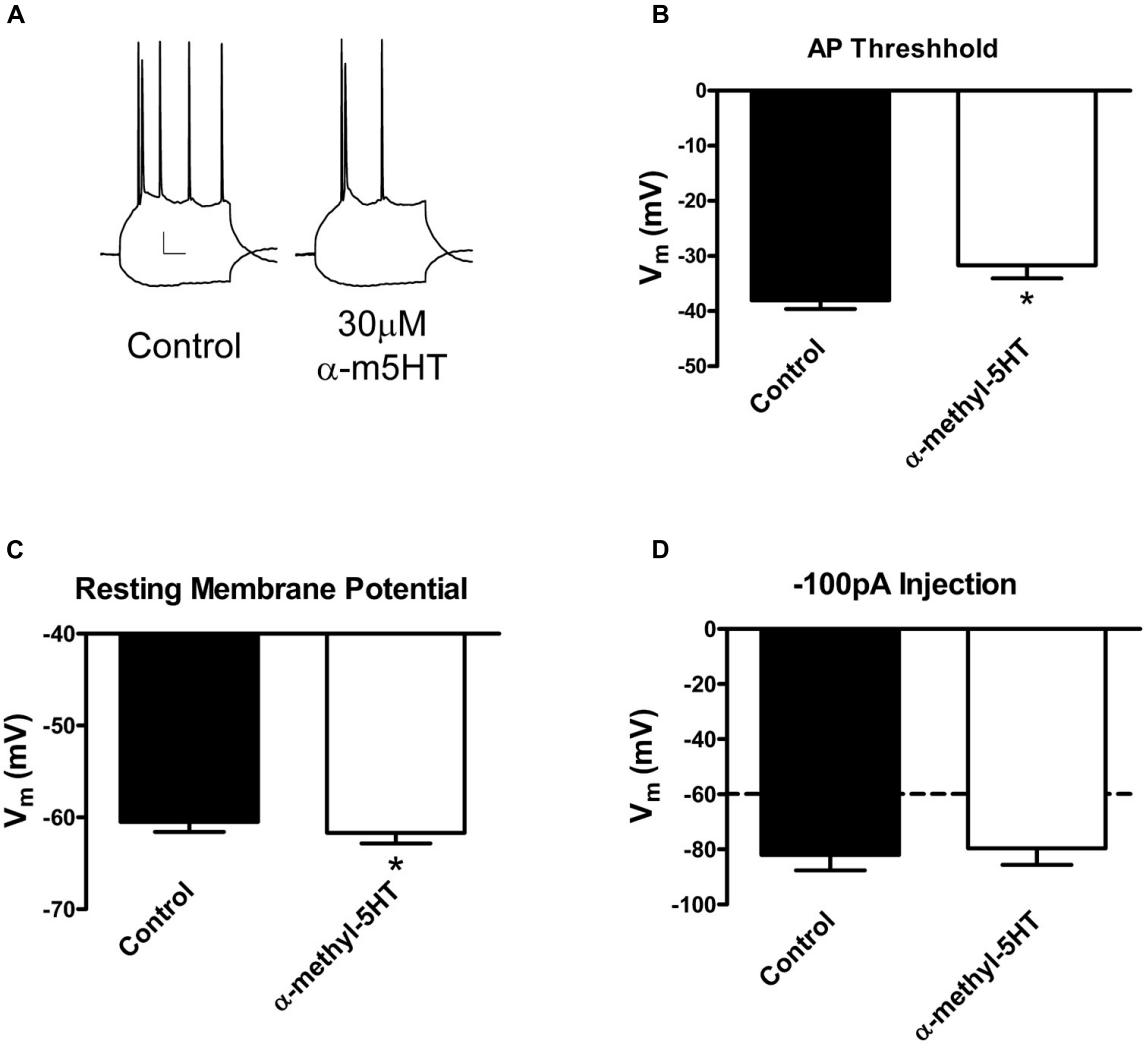
**α-methyl-5HT suppresses BLA principal neuron excitability via increasing threshold for action potential initiation.** **(A)** Exemplar traces illustrating the decrease in excitability following α-m5HT application in a BLA principal neuron. The traces were generated with identical hyperpolarizing and depolarizing current injections under control (standard aCSF) and ~10 min after drug treatment. Calibration bars: *x* = 10 ms, *y* = 10 mV. **(B)** α-m5HT significantly increased the membrane potential threshold required to generate an action potential in BLA principal neurons. *P < 0.01, paired *t*-test, *n* = 8. **(C)** α-m5HT caused a modest but significant hyperpolarization of the resting membrane potential. *P < 0.05, paired *t*-test. **(D)** α-m5HT did not alter the membrane response to a hyperpolarizing current injection.

Previous studies have shown that 5-HT2A/C receptors do not appear to modulate glutamatergic synapses in this brain region (Cheng et al., 1998). Our PS-EPSP findings show that the GABA_A_ antagonist bicuculline does not diminish α-m5HT modulation of BLA PS-EPSP suggesting that additional mechanisms may be important. We subsequently performed whole-cell current clamp recordings of BLA principal neurons to test the effects of the 5-HT2A/C agonist on excitability. We found that α-m5HT reduced the number of action potentials generated at moderate membrane depolarizations (**Figure 4A**). This was accompanied by a small but statistically significant hyperpolarization of the membrane potential from -60.5 ± 1.1 to -61.7 ± 1.2 mV (**Figure 4B**; *P* < 0.05 paired *t*-test, *n* = 6) as well as by an agonist-dependent increase in the membrane potential required to reach action potential threshold from -38.0 ± 1.6 to -31.7 ± 2.4 mV (**Figure 4C**; *P* < 0.05, paired *t*-test). This increase in action potential threshold was also accompanied by an α-m5HT-dependent broadening of the action potential, with area increasing from 280.3 ± 11.9 to 339.3 ± 29.4 mV × ms; but this effect did not reach statistical significant (not shown, *P* < 0.1, paired *t*-test). α-m5HT did not diminish the maximal number of action potentials that could be generated during a prolonged/strong depolarization (not shown) nor did it significantly alter the membrane response to a hyperpolarizing current injection (**Figure 4D**; *P* > 0.05, paired *t*-test). These data suggest that the 5HT2A/C-mediated inhibition of the BLA PS-EPSP is at least partially mediated by direct inhibition of BLA neuron excitability via an increase in action potential threshold.

## DISCUSSION

Microinjection of α-m5HT into the BLA prior to operant self-administration of a sweetened ethanol solution dramatically decreased the total number of responses during a non-reinforced “seeking” session. This inhibition of seeking is characterized by a significant decrease in the total number of responding bouts (continuous lever pressing without a pause > 20 s) without any alteration of the number of lever-presses per individual bout. These characteristics suggest that intra-BLA α-m5HT interferes with the initiation of a response bout rather than the processes that govern duration of a response bout once it has began. We cannot directly rule out α-m5HT-induced suppression of locomotor activity as potentially contributing to decreased lever-pressing behaviors. However, the selective effects on lever-pressing versus consumption (see below) suggest α-m5HT was not markedly sedating. Previous work has also shown that intra-BLA administration of a 5HT2 agonist does not affect closed-arm entries in the elevated plus maze (Cornelio and Nunes-de-Souza, 2007) and that intra-BLA depletion of serotonin had no impact on the total number of arm in this same assay (Sommer et al., 2001). Both measures have been consistently used in the literature as indirect indicators of locomotor behavior. This suggests that locomotor impairment may not be a significant contributor to the effects of α-m5HT on lever pressing behaviors.

Although the procedural separation of seeking- and drinking-behaviors is not common in the literature, work from the labs of Dr. Hank Samson and Dr. Cristine Czachowski permit some comparisons between BLA-dependent effects on lever pressing and other brain regions. For example, microinjection of the D2 dopamine receptor antagonist raclopride into the nucleus accumbens increased the latency to respond in non-reinforced sessions and also decreased the number of responses during these sessions (Czachowski et al., 2001; Samson and Chappell, 2004). Serotonin type 1B receptors in the nucleus accumbens also modulate seeking behaviors during non-reinforced sessions in animals trained to self-administer both sucrose and ethanol (Czachowski, 2005). These studies, together with our findings, support recent work illustrating the importance of the BLA-accumbens circuit during cue-induced responding (Ishikawa et al., 2008; Stuber et al., 2011) and emphasize that this circuit can regulate the “seeking” aspects of some operant behaviors. While the precise mechanism remains to be established in the context of the current operant paradigm, BLA modulation risk/reward-related decision-making (Ghods-Sharifi et al., 2009; Zeeb and Winstanley, 2011) and reward valuation (Shiflett and Balleine, 2010; Wassum et al., 2011) are both possible means by which BLA-dependent neurophysiology might regulate reward-seeking behaviors.

Compared to seeking behaviors, the effects of intra-BLA α-m5HT on drinking-related behaviors were more modest. Only the highest dose tested decreased the total amount of time animals spent licking the sweetened ethanol solution without any significant changes to the number of licks, lick bouts, or lick bout size. Since the total number of licks per session wasn’t altered by intra-BLA α-m5HT, the modest, non-significant effects on both the total number of lick bouts and the mean size of the lick bouts must have together summed to reduce the total time animals spent consuming the sweetened-ethanol solution. The BLA also modulates operant responding for food pellets but does not disrupt free-feeding in rats (Simmons and Neill, 2009). These findings suggest that the BLA exerts control over the “learned” seeking-consumption associations inherent within many operant paradigms. Regardless, the modest effects of intra-BLA α-m5HT on ethanol/sucrose consumption are very similar to the modest effects of intra-nucleus accumbens raclopride on ethanol drinking when employing the same model (Czachowski et al., 2001; Samson and Chappell, 2004). This nucleus accumbens serotonin system work also suggests that modulation of seeking and drinking behaviors may depend upon whether ethanol or sucrose is used as the reward (Czachowski, 2005). Reward-specific modulation in an operant setting appears to also generalize for limited-access to sucrose and ethanol in the home cage (Stratford and Wirtshafter, 2011). Since BLA-to-nucleus accumbens glutamatergic inputs convey primarily reward-related information (Pratt and Mizumori, 1998; Stuber et al., 2011), these data could suggest that modulation of the BLA by α-m5HT would be expected to influence primarily reward-seeking. But future comparisons between seeking/consuming behaviors directed at distinct rewards might help identify specific contributions by the BLA to these distinct but overlapping behavior outcomes in the operant setting.

Although the current study focused on ethanol-directed operant behaviors, the literature suggests that 5-HT2 receptors regulate many types of reward. For example, ketanserin modulates responding for food in both non-human primates (Brady and Barrett, 1985) and rodents (Higgins et al., 2013). 5-HT2 receptors can also modulate the discriminative and locomotor stimulant effects of both cocaine and nicotine in rodents (McMahon and Cunningham, 2001; Hayes et al., 2009; Nic Dhonnchadha et al., 2009; Higgins et al., 2013). In apparent contrast to these findings, Maurel et al. (1999) showed that 5-HT2 agonists specifically disrupt ethanol- but not water-directed operant behaviors in water-sated rats using a two-lever choice operant paradigm. Similarly, 5HT2 agonists disrupt reinstatement of operant responding for cocaine- but not sucrose-associated cues in food-sated animals (Burbassi and Cervo, 2008; Nic Dhonnchadha et al., 2009). These data together suggest that reward value may be critically important for 5HT2 receptor-mediated effects on operant behaviors. How might 5HT2-like receptors be involved in the recognition or reward value? These receptors modulate a complex array of animal behaviors in various models including anxiety (Kennett et al., 1994), impulsivity (Hadamitzky et al., 2009), and working memory (Terry et al., 2005). And BLA 5-HT2-like receptors specifically regulate anxiety-like behaviors (Zangrossi and Graeff, 1994), impulsive decision making (Hadamitzky and Koch, 2009), and conditioned aversion (Macedo et al., 2007). Given that acute ethanol is often considered an aversive stimulus in naïve animals (Phillips et al., 2005), complex interactions between anxiety, impulsive decision making, and conditioned behaviors may drive BLA-dependent modulation of operant behaviors directed at either drug or natural rewards. We should note that we cannot exclude potential effects of the self-administration procedure itself on BLA 5-HT2-like receptor expression or function. However, previous work using robust, non-contingent ethanol exposure (Ulrichsen, 1991; Pandey et al., 1992) or home-cage self-administration (Korpi et al., 1992) found very little impact on either receptor binding and/or signal transduction. Regardless, our data are consistent with the hypothesis that BLA 5-HT2-like receptors may be specifically involved with risk/reward evaluations in the context of operant self-administration. The current study also integrated behavioral studies with *in vitro* electrophysiology to better define potential neurophysiological mechanisms conferring the behavioral effects of intra-BLA α-m5HT. We specifically examined the effects of α-m5HT on BLA population spike/field EPSPs *in vitro* where it potently reduced PS-EPSP slope and amplitude. This effect was blocked by the 5-HT2 receptor antagonist ketanserin. α-m5HT and ketanserin are relatively selective for rat 5-HT2 receptors over other kinds of serotonin receptors and 5-HT2B receptors are not abundant in the central nervous system (Pompeiano et al., 1994). Although ketanserin has modest affinity for α1 adrenergic and histamine H1 receptors, our combined data are most consistent with the involvement of 5-HT2A or 5-HT2C receptors in the modulation of BLA synaptic responses. As for the specific neural mechanisms that might be involved, prior studies have shown that α-m5HTdepolarizes parvalbumin-positive local interneurons and stimulates spontaneous GABAergic neurotransmission onto the BLA principal glutamatergic projection neurons (Rainnie, 1999; Jiang et al., 2009). This suggested to us that 5-HT2A/C-stimulated release of GABA and subsequent activation of principal neuron GABA receptors might be a primary mechanism for any behavioral effects of intra-BLA α-m5HT microinjection. However, α-m5HT modulation of the BLA PS-EPSP was not dramatically attenuated by the GABA_A_ receptor antagonist bicuculline. One possible explanation is that α-m5HT-facilitated release of GABA may be robust enough to activate inhibitory GABA_B_ receptors which can hyperpolarize principal neurons directly (Washburn and Moises, 1992) or can act as presynaptic heteroreceptors to suppress release from glutamatergic afferents (Pan et al., 2009). GABA_B_ receptor activation at either cellular site would be sufficient to suppress the PS-EPSP measured in this study. Alternatively, 5-HT2 modulation of GABAergic interneurons may not be well represented in peak and slope portions of the BLA fEPSP. Previous work in the BLA has demonstrated 5-HT2 facilitation of spontaneous GABAergic synaptic activity (Rainnie, 1999; Jiang et al., 2009), and these events are dominated byperisomatic GABAergic synapses arising from feedback-type interneurons scattered throughout the brain region (McDonald, 1985; Silberman et al., 2010; Diaz et al., 2011). These feedback interneurons would not be strongly activated by a focal electrical stimulus like the kind we used to evoke the field EPSP. Rather, their synchronized activation would tend to occur after principal neuron activation during an experimentally evoked response. This synaptic architecture combined with the type of electrophysiological measures we used would under-represent any contributions by feed-back GABAergic interneurons in our measures. It is likely then that 5-HT2 facilitation of GABAergic mechanisms and direct inhibition of principal neuron excitability would both contribute to the behavioral pharmacology of intra-BLA α-m5HT. In this context, it is also noteworthy that 5-HT2A receptors are not strictly localized to GABAergic interneurons but are also expressed by BLA principal neurons (Jiang et al., 2009; Bombardi, 2011). 5-HT2C receptors are likewise expressed in the BLA (Li et al., 2004) although their cellular distribution has not been as well studied.

Serotonin has complex direct effects on principal neuron membrane potential that are mimicked by α-m5HT (Rainnie, 1999). Thus α-m5HT modulation of the PS-EPSP may also be consistent with direct modulation of principal neuron excitability by 5-HT2A/C receptors. We in fact found that α-m5HT reduced BLA neuron excitability by increasing action potential threshold and causing a small hyperpolarization of the resting membrane potential consistent with previous findings (Rainnie, 1999). Further, α-m5HTmodestly prolonged the action potential itself, which might enhance the inactivation of voltage-gated sodium channels to further inhibit BLA neuron excitability. Unfortunately, the molecular mechanisms mediating α-m5HT modulation of BLA action potential threshold and membrane hyperpolarization have yet to be established. And the intracellular signaling pathways engaged by Gq-coupled 5-HT2 receptors in the BLA have not yet been characterized. 5-HT2 receptor-mediated activation of various protein kinase signaling pathways are potential candidates. For example, PKC and PKA activity can inhibit neuron excitability via inhibition of voltage-gated sodium channel function (Numann et al., 1991; Cantrell et al., 2002) and in the case of PKC can increase action potential threshold (Dai et al., 2009). Regardless, our findings are consistent with the hypothesis that 5-HT2 receptors directly modulate the excitability of BLA pyramidal neurons. This mechanism, along with robust activation of GABAergic neurotransmission (Rainnie, 1999; Jiang et al., 2009), helps explain the robust effects of α-m5HT on BLA extracellular PS-EPSPs.

In summary, we have shown that microinjection of α-m5HT dramatically modulates seeking-, and to a lesser extent consumption-related, behaviors directed at a sweetened ethanol solution. We specifically employed a self-administration paradigm that procedurally separates seeking from consumption; and our findings illustrate the important role played by BLA activity in this self-administration paradigm. Our *in vitro* neurophysiology investigations suggest that 5-HT2 modulation of BLA principal neuron activity may be an important factor for these behavior outcomes. Our results also highlight the growing list of behavioral roles for BLA 5-HT2 receptors including the control of impulsive actions (Hadamitzky and Koch, 2009), innate anxiety-like behaviors (Leite-Panissi et al., 2006), and conditioned fear-like behaviors (Macedo et al., 2007). Future studies might employ 5-HT2A/C-focused approaches to determine the relative contributions of these specific behaviors to BLA-dependent reward-seeking.

## AUTHOR CONTRIBUTIONS

Daniel T. Christian and Jonathan A. Fetzer performed the *in vitro* neurophysiology experiments. Ann M. Chappell performed the behavioral pharmacology experiments. Daniel T. Christian helped with the manuscript. Brian A. McCool performed *in vitro* neurophysiology experiments, wrote the manuscript, and acted in a supervisory role.

## Conflict of Interest Statement

The authors declare that the research was conducted in the absence of any commercial or financial relationships that could be construed as a potential conflict of interest.
